# Perceptions of Family Physicians About Applying AI in Primary Health Care: Case Study From a Premier Health Care Organization

**DOI:** 10.2196/40781

**Published:** 2024-04-17

**Authors:** Muhammad Atif Waheed, Lu Liu

**Affiliations:** 1 Primary Health Care Corporation Doha Qatar; 2 Bath Business School Bath Spa University Bath United Kingdom

**Keywords:** AI, artificial intelligence, perception, attitude, opinion, surveys and questionnaires, family physician, primary care, health care service provider, health care professional, ethical, AI decision-making, AI challenges

## Abstract

**Background:**

The COVID-19 pandemic has led to the rapid proliferation of artificial intelligence (AI), which was not previously anticipated; this is an unforeseen development. The use of AI in health care settings is increasing, as it proves to be a promising tool for transforming health care systems, improving operational and business processes, and efficiently simplifying health care tasks for family physicians and health care administrators. Therefore, it is necessary to assess the perspective of family physicians on AI and its impact on their job roles.

**Objective:**

This study aims to determine the impact of AI on the management and practices of Qatar’s Primary Health Care Corporation (PHCC) in improving health care tasks and service delivery. Furthermore, it seeks to evaluate the impact of AI on family physicians’ job roles, including associated risks and ethical ramifications from their perspective.

**Methods:**

We conducted a cross-sectional survey and sent a web-based questionnaire survey link to 724 practicing family physicians at the PHCC. In total, we received 102 eligible responses.

**Results:**

Of the 102 respondents, 72 (70.6%) were men and 94 (92.2%) were aged between 35 and 54 years. In addition, 58 (56.9%) of the 102 respondents were consultants. The overall awareness of AI was 80 (78.4%) out of 102, with no difference between gender (*P*=.06) and age groups (*P*=.12). AI is perceived to play a positive role in improving health care practices at PHCC (*P*<.001), managing health care tasks (*P*<.001), and positively impacting health care service delivery (*P*<.001). Family physicians also perceived that their clinical, administrative, and opportunistic health care management roles were positively influenced by AI (*P*<.001). Furthermore, perceptions of family physicians indicate that AI improves operational and human resource management (*P*<.001), does not undermine patient-physician relationships (*P*<.001), and is not considered superior to human physicians in the clinical judgment process (*P*<.001). However, its inclusion is believed to decrease patient satisfaction (*P*<.001). AI decision-making and accountability were recognized as ethical risks, along with data protection and confidentiality. The optimism regarding using AI for future medical decisions was low among family physicians.

**Conclusions:**

This study indicated a positive perception among family physicians regarding AI integration into primary care settings. AI demonstrates significant potential for enhancing health care task management and overall service delivery at the PHCC. It augments family physicians’ roles without replacing them and proves beneficial for operational efficiency, human resource management, and public health during pandemics. While the implementation of AI is anticipated to bring benefits, the careful consideration of ethical, privacy, confidentiality, and patient-centric concerns is essential. These insights provide valuable guidance for the strategic integration of AI into health care systems, with a focus on maintaining high-quality patient care and addressing the multifaceted challenges that arise during this transformative process.

## Introduction

### Background

There is no universal definition for artificial intelligence (AI) [1]. AI has been defined in the literature as the branch of applied computer sciences in which algorithms are designed and are intended to perform different tasks while mimicking human intelligence [2]. It has been further defined as technologies that not only mimic human intelligence but can surpass them [3].

The global AI market in health care is projected to reach US $27.6 billion by 2025 [4]. One of the studies estimated that AI could save US $150 billion per annum by 2026 [5]. The human resource crisis in health care is already on the rise [6]. The global shortage of health care workers is approximately 17.4 million. Approximately, 50% of existing jobs will be in jeopardy or obsolete in 20 years [7].

Primary care is where AI would be most used in terms of opportunities on the broadest scale, where its power and future would be realized [8]. Moreover, AI has been regarded as a transformational force in the health care sector due to its impact on key stakeholders, such as primary care physicians, patients, systems, and financiers. AI will significantly impact many dimensions of clinical practice in the coming years, as machine learning (ML) and deep learning (DL) continue to hasten and will bring many advantages to both patients and clinicians [9]. There is not much literature on the impact of AI on employees, as little effort has been made empirically to study its impact [10]. Moreover, it is essential to know if AI is beneficial or detrimental to employees, as assisted AI, augmented AI, and autonomous AI have different implications on employee’s roles. Physicians are required to adjust their roles accordingly as AI is modeling practices nowadays, and if physicians fail to adjust their roles, it can lead to detrimental effects on overall patient care [11]. Physicians must be prepared to embrace the changes that AI will bring to their roles and to lead this change themselves. Furthermore, primary care physicians in health care are the main stakeholders and the most crucial, valuable, highly knowledgeable, and skilled human resource. Hence, it is essential to understand family physicians’ overall perception of AI application in primary care to develop organizational policies, modernize information technology infrastructure, develop AI literacy among physicians, establish and modify data privacy, data confidentiality, and code of ethics for the successful adoption and implementation of technology to gain a competitive advantage.

### Aims and Objectives

This study assessed the role of AI in the management and practices of the Primary Health Care Corporation (PHCC) in Qatar, emphasizing its fundamental potential in health care management. The primary objective was to evaluate the impact of AI in improving health care practice at the PHCC. In addition, the study sought to determine the role of AI in managing health care tasks and assess its impact on family physicians’ job roles. Moreover, this research also examined the challenges and ethical ramifications associated with introducing AI in primary care services at the PHCC.

### Literature Review

#### Understanding AI

AI is related to developing machines that mimic human cognitive processes such as learning, reasoning, and self-correction [3,12] and to performing tasks similar to a human mind [1]. It involves applying theoretical principles and the operation of applicable operating models to automate intellectual behaviors [13]. AI includes new concepts and solutions to address complex challenges [14]. In the field of medicine, AI introduces novel concepts such as a *digital physician*, reshaping the landscape [15].

#### Conceptualization of AI

The core of the AI system comprises neural-like elements, which are interconnected growing networks similar to a human brain that is active, associative, and homogenous with the ability to perceive, apprehend, and save information, enabling the system to learn, train, reason, and classify data to locate various patterns and connections to control external modalities [16]. To understand AI, it is essential to understand 2 key forms of AI: general and narrow AI. General AI refers to a machine’s ability to perform any intellectual task performed by a human, whereas narrow AI algorithms are designed for a limited task. Health applications using AI are generally of the narrow type. AI subfields in the health care sector are expert systems, automation of robotic processes, natural language processing, ML, and DL [6]. An example of an expert system is clinical decision support systems. The growth of AI in health care has been possible in recent decades due to the faster computer processing of data and data collection. Large amounts of data collection have been possible due to widespread electronic health records, mobile health, telehealth, and the Internet of Things. Improvements in natural language processing, ML, and DL have made AI possible up to the stage where it mimics human intelligence, fueling active discussion in the literature on whether AI can replace human doctors in the future [17].

#### AI and Health Care

The rapid growth of technology in health care has become a catalyst for evidence-based practice, and the integration of AI holds significant potential for improving health care service delivery [18]. The perceptions of AI’s impact, coupled with a deep understanding of the knowledge and interests of family physicians within primary care settings, is pivotal for the successful implementation of AI-based applications. Surprisingly, in an extensive survey across 4 of Saudi Arabia’s largest hospitals, a general lack of AI knowledge was evident among 250 doctors, nurses, and technicians [19]. This signifies the importance of addressing knowledge gaps to harness the benefits of AI effectively.

A gender-specific perspective on AI knowledge was highlighted in a study among 387 medical students in India, revealing that, although female students exhibited little initial AI knowledge, they displayed heightened interest in the field [20]. Moreover, AI was perceived to play a major role in health care service delivery in the future. This gender dimension adds nuance to the broader understanding of AI adoption in health care.

The projected role of AI in future health care service delivery is significant. Examples abound, such as AI therapy, which is a web-based course developed by the University of Sydney that uses cognitive behavioral therapy to help patients with social anxiety disorder [21]. In pathology, a DL-based convolution neural network achieved performance comparable with that of a human pathologist for detecting metastatic breast cancer in tissue slides from a lymph node biopsy. Similarly, the convolutional neural network–based system was more precise and accurate for a tissue slide–based scoring system to predict a decline in kidney function than a traditional pathologist [22]. In ophthalmology, the Food and Drug Administration–approved DL system has been used to detect diabetic retinopathy. Similarly, the DL system has been developed and evaluated to diagnose and classify cataracts in pediatric patients based on slit-lamp examination images, glaucoma based on retinal nerve fiber layer or visual field, and keratoconus based on Scheimpflug tonometry [22]. Physical robots are becoming more sophisticated as AI is incorporated into their operating systems and are likely to show the same intelligence level as other AI applications [23]. Moreover, surgical robots are also used in minimally invasive procedures such as in urological; gynecological; and ear, nose, and throat surgeries. In recent advances in AI applications, IBM’s Watson is an aiding tool for physicians to detect cardiovascular diseases and cancer [21]. The IBM Watson system can search and analyze data from a wide range of sources, surpassing human physicians’ capacity in knowledge [22]. Similarly, the picture archiving and communication system can detect signs of diseases from chest x-ray, ultrasound, magnetic resonance imaging, and computed tomography (CT) scan by contextualizing data from past images, clinical reports, and laboratory studies [24]. Opportunistic health care management is the provision of health services or interventions that are not planned but rather is an opportunity to address a health care need or an issue such as smoking cessation, screening for hypertension, prediabetes, and diabetes during a routine medical consultation. In the United Kingdom, for example, as a public health policy, “Making Every Contact Count” requires health care professionals to provide such interventions [25]. This role of the physician can be assisted by AI. AI can be used for opportunistic screening for diabetic retinopathy [26] and opportunistic screening of low bone density using contrast and noncontrast CT examinations [27]. AI algorithms identify minor or subclinical electrocardiogram abnormalities linked to a higher risk of developing left ventricular systolic dysfunction in the future [28]. During the COVID-19 pandemic, AI performed exceptionally well in the diagnosis, prognostic evaluation, epidemic forecasting, and drug discovery processes [29].

Building upon this extensive literature review, the following hypotheses were formulated:

- Hypothesis 1: perceived knowledge of AI among family physicians within the PHCC does not vary significantly based on age and gender.
- Hypothesis 2: family physicians at the PHCC perceive AI to have a positive impact on enhancing health care practices.
- Hypothesis 3: family physicians at the PHCC perceive AI to positively influence their roles in opportunistic health care management.

#### AI in Primary Care

The rapid advancement of AI technology has brought about transformative changes in health care management and has the potential to revolutionize various aspects of medical practice.

Kueper et al [30] conducted a scoping review of the literature on AI’s application in primary care, highlighting the evolving landscape of AI adoption. This study showed a shift from traditional expert systems to more sophisticated approaches, particularly supervised ML, mirroring the rapid advances in AI technologies. This paradigm shift holds profound significance for health care management, as AI gains increasing recognition as an asset in supporting health care professionals to make well-informed clinical decisions, especially when managing chronic conditions in high-income countries.

AI can assist with various health care tasks in primary care settings. AI predictive analytics tools have proven their efficacy in managing health care tasks [31]. These tasks include maintaining precise medical records, scheduling, inventory management, cost tracking, health promotion, clinical diagnosis, treatment planning, and developing care management plans. AI has the potential to enhance health care outcomes by streamlining operations within the health care system. Current academic literature presents an increasing trend in AI use in primary care and its positive effect on health care tasks, particularly in clinical management responsibilities [18]. The emergence of AI in electronic health record systems, which are extensively used today, has proven to be highly effective. AI-based clinical decision support systems have continued to evolve. Clinical decision support systems assist physicians and enhance patient safety by preventing dosage errors, drug duplication and presenting information on drug and drug interactions. Moreover, these systems helps physicians to adhere to clinical guidelines, order and interpret laboratory results, issue prompts and alerts for abnormal results, suggest follow-up actions, render treatment reminders and provide support for clinical and diagnostic coding [32]. Early diagnosis and treatment are essential for improving health outcomes. AI has shown its effectiveness in assisting doctors with image-based diagnoses of skin conditions and in proactively identifying patients at risk of developing dementia [15]. In England’s National Health Services, innovative applications of AI range from triage and symptom assessment to the automatic coding of clinical data, both in primary and community health care settings, thereby supporting personalized care management. This integration not only improves the quality of care but also saves valuable time for physicians, allowing them to focus more on providing personalized patient care. While the transformative potential of AI in clinical roles is evident, its impact on administrative functions has been relatively less explored [23]. Nevertheless, AI can play a vital role in optimizing administrative processes such as insurance collection, clinical reporting, medical billing, sales cycle management, and medical record management, ultimately contributing to more efficient health care operations and resource allocation. AI systems can perform routine operational tasks such as maintenance system management, accounting, and information inquiry much better and faster than human workers. AI-enabled chatbots and nursing robots can significantly improve operational process efficiency and reduce medical cost [33].

Collective evidence from the literature strongly suggests that AI holds promise as a constructive tool for managing various health care tasks at the PHCC. Its integration is expected to lead to improved clinical decision-making, operational efficiency, and ultimately contribute to enhanced patient care.

Accordingly, the following hypotheses were developed:

- Hypothesis 4: AI is perceived to play a constructive role in managing various health care tasks in PHCC.
- Hypothesis 5: family physicians at the PHCC perceive AI as having a positive impact on their clinical management responsibilities.
- Hypothesis 6: family physicians at the PHCC perceive AI as having a positive impact on their administrative management tasks.
- Hypothesis 7: AI is perceived to significantly improve the operational processes at the PHCC.

#### AI and Physicians

Whether AI will eventually replace physicians or complement them is still being debated, but it will significantly impact health care management activities and service delivery. The study by Ahuja [22] investigated whether AI will augment the physician’s role or eventually replace them using a quantitative survey methodology. The key finding was that AI would eventually replace radiologists in the field of radiology. This is because AI is more efficient and can handle and interpret millions of images in seconds. AI could interpret CT scans during the COVID-19 pandemic with 96% accuracy in just 20 seconds [34]. However, it has limitations, such as the inability to engage in complex interactions (ie, communication) with patients, failing to reassure patients, and to convey empathy. The study by Sarwar et al [35] concluded a positive attitude toward AI by taking the opinions of 487 pathologists from 54 countries regarding AI. However, the majority also had concerns regarding AI replacing their jobs. AI can triage cases as benign or malignant cases to pathologists, increasing diagnostic efficiency and accuracy and automated reporting, freeing 40% of pathologist time by reducing the workflow [36]. Esteva et al [37] conducted a comparative study testing 21 board-certified dermatologists against a convoluted neural network–trained system fed with 129,450 clinical images to the system. This study concluded that the CNN outperformed dermatologists in terms of both sensitivity and specificity. The study by Karches [38] argued that human physician judgment would remain better than that of AI in a primary care setting, as AI cannot adjust to recommendations according to individual patients’ needs. However, it cannot fine-tune its perception based on the patient’s history and examination, which appears to be a human-only ability. The study by Amisha et al [21] contends that machines cannot gather cues that only a physician can do during a patient-physician encounter. The machine cannot translate human traits, such as empathy, creativity, imagination, critical thinking, emotional intelligence, and interpersonal communication, both analytically and logically. According to the study by Meskó et al [39], the human physician is inevitable as empathy, communication, and human touch are included in the entire treatment process, which AI cannot provide; hence, AI will only be a helpful cognitive assistant. Physicians and nurses provide care to patients in an empathetic and compassionate environment that robotic physicians and nurses will not be able to do, as they lack the human characteristics of compassion [40]. Trust, empathy, and compassion are widely acknowledged as the core principles of effective health care [41]. Empathetic care enhances patient satisfaction.

Accordingly, the following hypotheses were developed.

- Hypothesis 8: the application of AI in health care tasks is perceived to lead to improved health care service delivery at the PHCC.
- Hypothesis 9: family physicians at the PHCC believe that AI is less likely to replace their current job roles.
- Hypothesis 10: family physicians at the PHCC perceive that AI decision-making does not surpass the judgment process of human physicians.
- Hypothesis 11: family physicians believe that the introduction of AI at the PHCC reduces patient satisfaction.

#### AI and Human Resource Management

AI is promising for closing the care gap in resource-poor settings, as digital health is widening. AI can address human resource shortages by ameliorating diagnostics, administrations, big analytics, and health care decisions [39]. AI and big data have significant impacts on strategic human resource management. Its digital transformation improves business processes [42] through the employee recruitment process (hiring and selection) and performance evaluation by providing real-time and accurate data and positively impacting staff retention [43]. AI is reported to reduce costs by providing evidence-based and affordable care to patients [39]. Furthermore, this will improve the overall quality of care. The actual economic impact of AI on health care is undetermined due to the methodological deficiencies in the literature analyzed in a systematic review [44]. Human resource management confers a competitive advantage through employment management by placing capable and highly committed workers and incorporating structural, cultural, and personal techniques [45]. Human resource management aims to manage human capital in modern organizations, which is the most vital asset. Instead of focusing on power, human resource management should invest more in employee training and development because it is a significant source of innovation and development [46]. Mutual complementation of humans and machines creates more value for the organization, as machines help data interpretation and analysis, and humans in innovation and social interaction. AI frees employees from repetitive tasks, but at the same time, it also needs the development of higher collaborative competencies among employees. Firms in the future would need new human resource plans with the need to develop policies by reviewing the need for structural changes and capacity models, and recent reforms would be required for enterprise human resource management. Furthermore, a negative attitude toward AI needs to be addressed among employees by properly engaging them and studying AI in depth [46].

Accordingly, the following hypothesis was developed:

- Hypothesis 12: the introduction of AI is perceived to assist and enhance human resource management practices at the PHCC.

#### AI Challenges, Risks, and Ethical Ramifications

The study by Laï et al [1] argued that there are many concerns regarding AI. These include the fuzzy notion of AI, health data confidentiality issues, growth in AI knowledge, international competition, and disruption of the patient-physician relationship. Furthermore, the diagnosis and decision-making landscape is expected to change for both physicians and patients, and these developments would impact the entire health care system. The implementation of AI will be another challenge, and AI must add value and should support and not subvert the patient-physician relationship, as health care is a social endeavor based on human interactions. If AI is implemented correctly, emotional and cognitive spaces would open for physicians; however, if implemented incorrectly, it will have severe consequences [8]. The existing information technology infrastructure might be outdated to adopt AI systems, which will require careful review before implementation. The adoption of AI-based technologies by resource constrained countries can be wider, as they will be more open to policy changes compared to resource-rich countries [39]. With the introduction of AI technology, the patient-physician relationship will change significantly. The hierarchy will still be in place and the patient-physician relationship will be more just than ever before but patients’ autonomy is still a question. Similarly, the development of standards for collecting data and testing, which stakeholders, clinicians, industry, and scientists should lead, will be challenging.

Accordingly, the following hypotheses were developed:

- Hypothesis 13: the implementation of AI is not expected to undermine the patient-physician relationship from the family physician’s perspective at the PHCC.
- Hypothesis 14: family physicians’ perceptions of challenges and ethical ramifications when introducing AI at the PHCC do not significantly differ based on age and gender.

## Methods

### Overview

This study adopted a cross-sectional research design, using a quantitative approach. The primary focus of a quantitative methodology is to identify the relationship between variables and to accept or reject connections or linkages between these variables [47]. Moreover, it reduces bias probabilities, as the researcher is independent of the respondents, both physically and emotionally, and establishes standardization of investigation and interpretation rather than situational analysis.

### Participants and Data Collection

The PHCC in Qatar is the main provider of primary health care services via its 28 health centers, scattered across all regions of Qatar. There are 724 family physicians working in the organization. On the basis of a CI of 95%, an expected proportion of 0.5, and a margin of error of 5, the sample size was 252. The questionnaire was sent via PHCC intranet email by the operations department of the PHCC to all family physicians for 4 weeks in March 2021, with reminder emails sent after the second and third week. A total of 132 physicians participated in the study. Among them, 102 questionnaires were fully completed that were eligible for data analysis. Incomplete questionnaires were not included because all questions were eligible for the hypothesis test. The response rate was 14.1% (102/724).

The demographic characteristics of participants who were early and late responders were analyzed by splitting the data into 2 groups based on the date of response. The analysis showed that early responders were similar to late responders in age (*P*=.15), gender (*P*=.99), working status (*P*=.33), and licensed years (*P*=.11). Although the response rate was low, it can be assumed that the nonresponse bias was minimal.

### Data Analysis

Data collected from participants using Survey Monkey software (Symphony Technology Group) were transferred to SPSS (version 27; IBM Corp) for statistical analysis. The information was codified using the data statistics editor in SPSS. The Likert scale had 5 categories: strongly agree, agree, neutral, disagree, and strongly disagree. The Likert scale data were forward scored on a numeric scale of 1 to 5 to facilitate statistical analysis.

Descriptive and inferential statistical models were used to analyze the survey results. Nonparametric tests were chosen because the data were not normally distributed, as confirmed using the Kolmogorov test. The Shapiro-Wilk test is commonly used for sample sizes <50, while the Kolmogorov approach is used for sample sizes >50 to assess the normality of the data distribution [48].

The Spearman rho correlation coefficient, which assesses the 2-way linear relationship between 2 variables, was used to determine whether the application of AI in health care tasks is perceived to lead to improved health care service delivery at PHCC. The Spearman rho correlation value ranges from +1 to –1, where 0 represents no relationship, +1 indicates a perfect positive correlation, and –1 indicates a perfect negative correlation [49].

The chi-square goodness-of-fit test, comparing expected and observed values in categorical variables [50], was used to assess family physicians’ perceptions of AI’s role in job replacement, human resource management, and patient-physician relationships. The chi-square test of homogeneity, comparing proportions between ≥2 groups [51], was used to examine variations in AI knowledge and challenges, as well as ethical ramifications by age and gender. To compare column proportions by age and gender groups, multiple corrections were made using the Bonferroni correction.

The 1-sample Wilcoxon test was used to assess perceptions of AI’s positive impact on health care practices, clinical and administrative tasks, and patient satisfaction at the PHCC. This test, an alternative to the standard 1-sample *t* test, is assumed to be more sensitive to the sign test, measuring positive and negative ranks for testing significance using the hypothesized median set as neutral (0) when testing these hypotheses [52].

### Validation

The survey questionnaire, comprising 47 questions, underwent a systematic process of piloting, testing, and validation to eliminate potential ambiguity for the respondents. Primarily using the Likert scale, it covered five main constructs: (1) demographics (4 items), exclusively designed to capture participant data without internal consistency measurement; (2) family physician’s knowledge and perspective on clinical management of AI (11 items, Cronbach α=0.873); (3) family physician perspective on administrative management of AI (10 items, Cronbach α=0.916); (4) family physician’s perspective on public health management of AI (9 items, Cronbach α=0.930); and (5) family physicians’ perspectives on AI challenges, ethical ramifications, and impact on job roles (13 items, Cronbach α=0.744). The overall Cronbach α score for the research instrument, excluding demographics, was 0.937, indicating exceptional reliability per the established standard [53]. The respondents took mean time of 12 (SD 9) minutes to complete the survey.

### Ethical Considerations

This paper was developed from the first author’s (MAW’s) dissertation that he completed with the University of Liverpool in partial fulfillment of the requirements for a master’s degree when the second author (LL) was the supervisor. The original research project was approved by both the University of Liverpool, United Kingdom Research Ethics Committee, and PHCC, Qatar Research Subcommittee (approval no: PHCC/DCR/2020/07/079). Permission was obtained by sending a questionnaire via the intranet email from the organization. In the web-based survey questionnaire, an initial page was provided to participants to explain the nature, purposes, and expected duration of the research. Moreover, it was ensured to the participants that this study was entirely voluntary, their data would be dealt with in the strictest confidential manner, and no information would be collected to identify them.

## Results

### Descriptive Statistics

Descriptive statistics were used to summarize the research findings using the frequency and percentage of responses. The responses on the Likert scale were collapsed and recategorized into 3 main groups: agree, neutral, and disagreed.

### Demographic Data

The demographic data are summarized in Table 1. The majority of respondents (72/102, 70.6%) were men, 94 (92.2%) out of 102 were in the age group of 35 to 54 years, and 58 (56.9%) out of 102 worked as consultants.

**Table 1:** Participants’ demographic data (N=102).

Characteristics		Values, n (%)
**Age groups (years)**		
	25-34	1 (1)
	35-44	44 (43.1)
	45-54	49 (48)
	55-64	8 (7.8)
**Gender**		
	Men	72 (70.6)
	Women	30 (29.4)
**Working status**		
	General practitioner	27 (26.5)
	Pediatrician	1 (1)
	Associate specialist	7 (6.9)
	Consultant	58 (56.9)
	Senior consultant	7 (6.9)
	Manager	1 (1)
	Executive	1 (1)
**Licensed years**		
	1-2	2 (2)
	2-5	16 (15.7)
	5-10	29 (28.4)
	10-20	31 (30.4)
	20-30	19 (18.6)
	30-40	5 (4.90)

### Perceived Knowledge of AI

Of the 102 family physicians surveyed for AI awareness, 7 (6.9%) out of 102 were extremely aware, 18 (17.6%) out of 102 were very aware, 55 (53.9%) out of 102 were somewhat aware, 20 (19.6%) out of 102 were not so aware, and 2 (2%) out of 102 had no awareness. Overall, AI awareness among PHCC physicians was 78.4% (80/102). The results are summarized in Table 2.

**Table 2:** Perceived knowledge of artificial intelligence (AI; N=102).

Perceived knowledge of AI	Values n (%)
Extremely aware	7 (6.9)
Very aware	18 (17.6)
Somewhat aware	55 (53.9)
Not so aware	20 (19.6)
Not at all aware	2 (2)
Overall awareness	80 (78.4)

### Family Physicians’ Perspective on Clinical and Administrative Role of AI in Health Care Management

Table 3 depicts the perspective of family physicians on the clinical and administrative role of AI in health care management. Most of the respondents (73/102, 71.6%) acknowledge the potential of AI in triage, while 60 (58.8%) out of 102 believe in its efficacy for assisting in emergency case management. Regarding clinical assessment and diagnostic management tasks, 69.6% (71/102) agree with the assistive role of AI, with 55 (53.9%) out of 102 of physicians foreseeing its capability to surpass conventional methods of diagnostic report management. Furthermore, 81 (79.4%) and 56 (54.9%) out of 102 of physicians believe in AI's assistance in medication management requirements and improving patient treatment compliance, respectively. On the administrative role of AI, most physicians (86/102, 84.3%) perceive AI as managing health care performance by enhancing information dissemination, while 78 (76.5%) out of 102 anticipate improved efficiency in health care administrative activities. The positive perceptions extend to the care management systems, with 83 (81.4%) out of 102 agreeing on AI’s improving them and 79 (77.5%) out of 102 endorsing its ability to reduce medical errors. This collective optimism highlights the potential transformative impact of AI in enhancing the clinical and administrative roles of family physicians and, hence, health care delivery.

**Table 3:** Family physicians’ perspective on the clinical and administrative management role of artificial intelligence (AI; N=102).

		Agree, n (%)	Neutral, n (%)	Disagree, n (%)
**AI on clinical management**				
	AI can assist in triage	73 (71.6)	23 (22.5)	6 (5.9)
	AI can assist in managing emergency cases	60 (58.8)	27 (26.5)	15 (14.7)
	AI will assist in clinical assessment and diagnosis management tasks easy	71 (69.6)	24 (23.5)	7 (6.9)
	AI will supersede the conventional methods of diagnostic reports management	55 (53.9)	30 (29.4)	17 (16.7)
	AI will improve clinical judgment process	65 (63.7)	28 (27.5)	9 (8.8)
	AI has the potential for the task management and clinical investigation and data storage	86 (84.3)	15 (14.7)	1 (1)
	AI will assist to follow clinical pathways	82 (80.4)	19 (18.6)	1 (1)
	AI will assist in medication management requirements	81 (79.4)	19 (18.6)	2 (2)
	AI integration will make patients treatment compliance better	56 (54.9)	35 (34.3)	11 (10.8)
	AI enhances overall management of patient care	71 (69.6)	29 (28.4)	2 (2)
**AI on administrative management**				
	AI integration will help in improving care management systems	83 (81.4)	18 (17.6)	1 (1)
	AI can make effective plans to reduce medical errors	79 (77.5)	22 (21.6)	1 (1)
	AI will help in managing health care performance by improving information dissemination	86 (84.3)	15 (14.7)	1 (1)
	AI will be more helpful in management of service provision	78 (76.5)	22 (21.6)	2 (2)
	AI integration will make health care administrative activities more robust and successful	78 (76.5)	20 (19.6)	4 (3.9)
	AI helps in financial planning and management	75 (73.5)	24 (23.5)	3 (2.9)
	AI assists in health care policy making	67 (65.7)	24 (23.5)	11 (10.8)
	AI has potential for planning treatment care pathways	73 (71.6)	23 (22.5)	6 (5.9)
	AI will assist human resource management (recruitment and retention)	65 (63.7)	29 (28.4)	8 (7.8)
	AI introduction will be advantageous to administrative staff	74 (72.5)	23 (22.5)	5 (4.9)

### Family Physicians’ Perspective on the Role of AI in Public Health Management

Table 4 presents the family physicians’ perspectives on the role of AI in public health management. Family physicians overwhelmingly supported the integration of AI in public health, with 80 (78.4%) out of 102 respondents endorsing its role in organizing tasks for public health awareness and 84 (82.4%) out of 102 endorsing its role in managing public health surveillance. Interestingly, 85 (83.3%) out of 102 agreed on AI’s efficacy in providing disease reports for disease prediction and management. A significant majority (86/102, 84.3%) perceived AI as a valuable tool for opportunistic health care screening. Moreover, 78 (76.5%) out of 102 believed in AI’s effectiveness during epidemics and 72 (70.6%) out of 102 agreed that it aids in managing health care logistics and reducing costs during pandemics. These findings highlight the positive perception of AI’s multifaceted benefits in enhancing public health strategies and outcomes.

**Table 4:** Family physicians’ perspectives on the role of artificial intelligence (AI) in public health management (N=102).

		Agree, n (%)	Neutral, n (%)	Disagree, n (%)
**AI on public health management**				
	AI is beneficial for organizing tasks for public health awareness	80 (78.4)	21 (20.6)	1 (1)
	AI helps in disease screening and monitoring	84 (82.4)	17 (16.7)	1 (1)
	AI is an efficient tool for assessing and managing risks to public health	80 (78.4)	20 (19.6)	2 (2)
	AI has the potential for providing reports for disease prediction and disease management	85 (83.3)	17 (16.7)	0 (0)
	AI may be considered by physicians as a beneficial tool in managing public health surveillance	84 (82.4)	18 (17.6)	0 (0)
	AI introduction in health care management will make opportunistic health care screening easier	86 (84.3)	16 (15.7)	0 (0)
	AI is effective tool in managing quality of care in epidemics	78 (76.5)	21 (20.6)	3 (2.9)
	AI is an efficient tool in disease containment projects planning	73 (71.6)	27 (26.5)	2 (2)
	AI will help in managing health care logistics and reduce cost during pandemics	72 (70.6)	30 (29.4)	0 (0)

### Family Physicians’ Perspective on AI Challenges and Ethical Ramifications in Health Care and Impact on Their Job Roles

Table 5 shows that family physicians expressed concerns about AI challenges, ethical ramifications in health care, and their impact on their job roles. A majority (61/102, 59.8%) worried about patient confidentiality due to potential hacking of AI-managed health care records; similarly, 61 (59.8%) out of 102 were concerned about the risk to organizations’ confidential data. Regarding decision-making, 69 (67.6%) out of 102 acknowledged potential conflicts with humans due to differences in decision-making and 80 (78.4%) out of 102 expressed concern about AI lacking emotional input. Patient satisfaction was a concern for 76 (74.5%) out of 102 due to the absence of emotions in AI-driven decisions. In addition, 65 (63.7%) out of 102 believed AI’s clinical judgment may be inferior to that of physicians. While 42 (41.2%) out of 102 agreed AI could be accountable in malpractice cases, 89 (87.3%) out of 102 emphasized the need for AI training for health care managers and staff. However, 33 (32.4%) out of 102 found learning AI challenging for health care staff. Family physicians expressed nuanced views on AI’s impact on their roles. The majority (74/102, 72.5%) believed that AI cannot replace their jobs, with 53 (52%) out of 102 asserting that it will not undermine the patient-physician relationship. A total of 35 (34.3%) out of 102 were open to using AI in medical decisions in the future. These findings demonstrated family physicians’ perceived AI risks, such as data privacy, confidentiality, the decision-making process of AI, its accountability in cases of malpractice, and the need for training to learn AI. Moreover, it also highlighted a balanced perspective on AI’s role, emphasizing AI augmenting the roles of family physicians rather than replacing them.

**Table 5:** Family physicians’ perception on artificial intelligence (AI) challenges and ethical ramifications and impact on their job role (N=102).

		Agree, n (%)	Neutral, n (%)	Disagree, n (%)
**AI challenges and ethical ramifications**				
	Management of health care records through AI may threaten patient confidentiality due to hacking	61 (59.8)	32 (31.4)	9 (8.8)
	Management through AI may threaten health care organizations confidential data due to hacking	61 (59.8)	30 (29.4)	11 (10.8)
	Management of health care operations involving AI may conflict with humans due to difference in decision-making	69 (67.6)	26 (25.5)	7 (6.9)
	Decision-making process by AI in health care encounters lacks emotional input	80 (78.4)	16 (15.7)	6 (5.9)
	Management of decision-making process through AI may decrease patient satisfaction due to lack of emotions	76 (74.5)	18 (17.6)	8 (7.8)
	Patients’ satisfaction is decreased with inclusion of AI in decision-making process management	47 (46.1)	42 (41.2)	13 (12.7)
	Process of clinical judgment by AI might be inferior to that made by physicians	65 (63.7)	26 (25.5)	11 (10.8)
	In case of malpractice AI integration in decision-making process can be held accountable	42 (41.2)	39 (38.2)	21 (20.6)
	Health care managers and staff will require training in AI-based operations	89 (87.3)	12 (11.8)	1 (1)
	Management of health care processes through AI are hard to learn for health care staff	33 (32.4)	40 (39.2)	29 (28.4)
	AI could not replace physician job	74 (72.5)	17 (16.7)	11 (10.8)
	AI would not undermine patient-physician relationship	53 (52.0)	28 (27.5)	21 (20.6)
	AI will be used in making medical decision in future	35 (34.3)	43 (43.1)	23 (22.5)

### Hypothesis

Table 6 illustrates a summary of the hypotheses tested, the statistical tests used, corresponding *P* values and key findings with their relevant implications. The perceived knowledge of AI among different age and gender groups (hypothesis 1) examined by using the chi-square test of homogeneity showed no statistical significance for the perceived knowledge of AI among family physicians within the PHCC based on age and gender groups. The awareness of the physicians who were men was (60/72, 83%), and that of the women awareness was (20/30, 67%; *P*=.06). Similarly, regarding the awareness of AI between physicians aged 18 to 54 years (72/94, 77%) and aged >55 years (8/8, 100%) with *P*=.12. Licensed years and working status also had no statistical significance with awareness of AI (*P*=.50 and *P*=.51, respectively). Chi-square tests of homogeneity showed no significant differences across age and gender groups regarding 10 item, AI challenges and ethical ramifications (hypothesis 14; *P*>.05). A 1-sample Wilcoxon signed-rank test confirmed a perceived positive role of AI in health care practice, task management, and operational processes at PHCC (hypotheses 2, 4, and 7; *P*<.001). In addition, a Spearman rho test demonstrated a moderate to strong correlation between health care tasks and health care service delivery (hypothesis 8; Spearman rho=0.679, *P*<.001). The analyses using a 1-sample Wilcoxon signed-rank test further supported the positive impact of AI on family physician opportunistic health and clinical and administrative roles (hypotheses 3, 5, and 6; *P*<.001), while anticipating a reduction in patient satisfaction (hypothesis 11; *P*<.001). Importantly, the results indicated that AI is not expected to negatively impact the patient-physician relationship (hypothesis 13; *P*<.001) and will not replace human physicians (hypothesis 11; *P*<.001). These findings provide valuable insights into the strategic integration of AI into health care settings.

**Table 6:** Summary of hypothesis testing using specific statistical tests (chi-square test of homogeneity and goodness-of-fit, 1-sample Wilcoxon signed-rank test, and Spearman rho), corresponding *P* values, key findings and their implications.

Hypothesis	Statistical test	*P* value	Key findings and implications
Hypothesis 1: perceived knowledge of AI^a^ among family physicians within the PHCC^b^ does not significantly vary based on age and gender groups.	Chi-square test of homogeneity	.12 for age*;* .06 for gender groups	No significant difference in AI perceived knowledge across age and gender groups.
Hypothesis 2: family physicians at the PHCC perceive AI to have a positive impact on enhancing health care practices.	1-sample Wilcoxon signed-rank test	<.001	Strong evidence is supporting the perceived positive role of AI in health care practice.
Hypothesis 3: family physicians at the PHCC perceive AI to positively influence their roles in opportunistic health care management.	1-sample Wilcoxon signed-rank test	<.001	Affirms the perceived positive influence of AI on opportunistic health care management roles.
Hypothesis 4: AI is perceived to play a constructive role in managing various health care tasks at the PHCC.	1-sample Wilcoxon signed-rank test	<.001	Strong evidence suggesting AI’s perceived beneficial impact on health care task management.
Hypothesis 5: family physicians at the PHCC perceive AI to have a positive impact on their clinical management responsibilities.	1-sample Wilcoxon signed-rank test	<.001	Indicates a perceived positive effect of AI on clinical management roles.
Hypothesis 6: family physicians at the PHCC perceive AI to have a positive impact on their administrative management tasks.	1-sample Wilcoxon signed-rank test	<.001	Provides evidence of AI’s perceived positive influence on administrative roles.
Hypothesis 7: AI is perceived to significantly improve the operational processes at the PHCC.	1-sample Wilcoxon signed-rank test	<.001	Strong evidence supporting AI’s perceived positive influence on health care operations.
Hypothesis 8: the application of AI in health care tasks is perceived to lead to improved health care service delivery at the PHCC.	Spearman rho test^c^	<.001	Moderate to strong positive correlation between perceived AI application in health care tasks and health care service delivery.
Hypothesis 9: family physicians at the PHCC believe that AI is less likely to replace their current job roles.	Chi-square goodness-of-fit test^d^	<.001	Strong evidence against the hypothesis of AI job replacement as perceived by family physicians.
Hypothesis 10: family physicians at the PHCC perceive that AI decision-making does not surpass the judgment process of human physicians.	One sample Wilcoxon signed-rank test	<.001	Strong evidence against the superiority of AI decision-making over human judgment as perceived by family physicians.
Hypothesis 11: the introduction of AI is believed to reduce patient satisfaction by family physicians at the PHCC.	One sample Wilcoxon signed-rank test	<.001	Strong evidence that AI has a negative impact on patient satisfaction as perceived by family physicians.
Hypothesis 12: the introduction of AI is perceived to assist and enhance human resource management practices at the PHCC.	Chi-square goodness-of-fit test^e^	<.001	Strong evidence supporting the idea that AI is perceived to assist in human resource management.
Hypothesis 13: the implementation of AI is not expected to undermine the patient-physician relationship from the family physician perspective of the PHCC.	Chi-square goodness-of-fit test^f^	<.001	Strong evidence against the hypothesis of AI is perceived to negatively impacting the patient-physician relationship.
Hypothesis 14: family physicians’ perceptions of challenges and ethical ramifications when introducing AI at the PHCC do not significantly differ based on age and gender.	Chi-square test of homogeneity	>.05	No significant differences in perceived challenges and ethical ramifications among age and gender groups.

^a^AI: artificial intelligence.

^b^PHCC: Primary Health Care Corporation.

^c^Correlation coefficient of health care tasks and health care service delivery was Spearman rho=0.679 (moderate to strong correlation).

^d^*χ*^2^_2_=71.1; N=102.

^e^*χ*^2^_2_=48.8; N=102.

^f^*χ*^2^_2_=16.6; N=102.

## Discussion

### Principal Findings

The primary findings of this study offer valuable insights into the perceptions of PHCC family physicians in Qatar regarding the integration of AI in the health care context. The overall awareness of AI among PHCC physicians in Qatar was 78.4% (80/102). Moreover, the proportion of physicians with very aware and extremely aware levels of AI was 24.5% (25/102), reflecting a robust understanding of AI technology. Critically, the statistical analysis did not reveal any meaningful variations in perceived AI knowledge based on gender (*P*=.06) or age groups (*P*=.12). Similarly, the exploration showed no statistically significant correlations between AI awareness and factors such as years of licensure (*P*=.50) or current working status (*P*=.51). Similarly, no significant disparities in perceived AI challenges and ethical implications were identified among physicians of diverse age and gender groups (*P*>.05). Furthermore, the results highlight the affirmative role that physicians perceive AI might play in the enhancement of health care practices at the PHCC (*P*<.001), facilitating improved management of health care tasks (*P*<.001), optimizing operational processes (*P*<.001), and fostering effective human resource management (*P*<.001). Notably, AI was perceived to exert a beneficial influence on the multifaceted roles of family physicians in clinical (*P*<.001), administrative (*P*<.001), and opportunistic health care management (*P*<.001). It is crucial to highlight that the study findings indicate physicians’ perception that AI decision-making does not supersede the clinical judgment process of human physicians (*P*<.001), and the introduction of AI is not anticipated to compromise the essential patient-physician relationship (*P*<.001). Moreover, from the perspective of family physicians, AI was less likely to displace their existing job roles (*P*<.001). However, the implementation of AI was expected to result in reduced patient satisfaction (*P*<.001).

### Comparison With Prior Work

The overall awareness of AI among PHCC physicians stands at 78.4% (80/102), reflecting a significant level of perceived knowledge. This heightened awareness may facilitate the implementation of AI without substantial resistance [54]. This awareness level is notably higher than that in the study conducted by Oh et al [55], where only 5.9% of Korean medical students and doctors perceived a strong familiarity with AI, despite Korea’s reputation as technologically advanced.

Consistent with the proposition found in the study by Lin et al [8], our findings indicate that PHCC physicians perceive AI as a transformative force in primary care. Importantly, our research affirms that from the physicians’ perspective, AI is less likely to replace the role of the family physician and does not surpass the human physician decision-making process. This aligns with the literature, which asserts that AI enhances the diagnostic capability of family physicians rather than replacing their diagnostic intelligence [56].

Our study demonstrates that PHCC physicians perceive AI as a valuable tool for human resource management, positively impacting both employee retention and recruitment, which is consistent with the literature. Despite being a relatively novel concept, AI has the potential to streamline recruitment processes, leading to more efficient and high-quality employee selection [57]. Furthermore, AI’s influence extends across key domains of human resource management, as indicated by its potential to enhance recruitment, placement, staff development, performance management, compensation management, human relations management, and strategic planning of human resources [58]. AI-based systems, such as those using automated recruitment tasks and reducing bias, hold promise for improving the efficiency and effectiveness of human resource functions.

The perception among PHCC physicians that AI improves operational processes and reduces the cost of care aligns with existing literature. Predictive analytics, including forecasting, enhance capacity management, resource use, and improvement in overall business processes, contributing to operational innovation in health care [59]. In addition, routine operational processes can be made quicker and more efficient through AI integration.

Although, nowadays, AI can demonstrate superior performance compared to physicians in certain specialties, such as dermatology (analysis of skin lesions), pathology (slide scanning), cardiology (electrocardiographic interpretation), and radiology (analysis of clinical images) [60], it is not perceived as surpassing the broader clinical decision-making process of human physicians. Patient satisfaction may be reduced due to AI’s limitations in replicating human characteristics, such as empathy, compassion, and human touch [61], and complete acceptance of fully automated services remains a challenge. Nevertheless, AI’s superiority in specialized domains underscores its potential to complement medical practitioners in specific areas.

This study highlights the perceived positive impact of AI on opportunistic health care management, which was evident particularly during the COVID-19 pandemic. The use of AI in tracking, prediction, contact tracing, early diagnosis, monitoring, and vaccine development highlights its crucial role in addressing pandemic health care challenges [62]. Approximately 36 countries have used AI- and ML-based applications for digital contact tracing to limit the spread of SARS-CoV-2 [63]. The Ministry of Public Health of Qatar has also adopted AI-based tools for contact tracing, and this exemplifies how AI can contribute to crisis management and safeguarding public health.

Given the perception of family physicians, this research establishes that AI integration positively affects PHCC service delivery, enhancing health care task management and care systems. AI will automate many administrative tasks where managers, administrators, and health care staff spend about 54% of their time on them [64]. Family physicians’ clinical and administrative roles may benefit from AI integration, reducing administrative burdens and allowing them to focus on patient-centered care, increasing their professional fulfillment and reducing burnout [65]. AI’s potential for disease prediction, digital health coaching, evidence-based clinical decisions, and medication management improvement holds promise for improving the quality of care provided.

PHCC physicians perceived ethical considerations surrounding AI, including informed consent, safety, transparency, biases, and data privacy, aligned with concerns found in the literature [66]. Notably, 41.2% (42/102) of participants in this study advocated AI’s liability in cases of malpractice, reflecting the need for robust accountability mechanisms. Recent regulatory updates, such as the introduction of the Medical Device Regulation in Europe, reflect the evolving legal landscape of AI [66]. Policy makers should consider product liability, deterrence, and compensation as they navigate this dynamic terrain.

While the impact of AI on patient-physician relationships remains uncertain [67], our study concludes that from the physicians’ perspective, AI will not subvert these relationships. However, careful and strategic planning is essential during AI implementation to prevent potential negative consequences. The balance between cost reduction, efficiency, and accuracy considerations while upholding patient-physician dynamics is of paramount importance.

### Limitations and Further Research

This study produced compelling findings and will serve as a springboard for future researchers to replicate similar studies. However, it is critical to understand the limitations of a study because they reflect flaws that could influence the outcomes and conclusions [68]. First, it used a positivist paradigm that limits family physicians’ richer perspectives in a broader context for applying AI in PHCC management and practices. Second, this study only included the PHCC, a single organization, and the response rate in this study was low despite sending 2 reminder emails to practicing family physicians at the PHCC. However, response rates have been declining in health care field–related surveys [69] and physicians’ response rates have continued to decline [70].

This research can be replicated based on an interpretivist paradigm and by using semistructured interviews to obtain deeper insights and richer knowledge about the perception of family physicians regarding the application of AI in a primary care setting. Perhaps using mixed methods will provide a deeper understanding and add more rigor to research regarding the application of AI in primary care [71]. Future research can also examine the factors that lead to resistance to AI implementation in primary care. Moreover, it should include nurses’ administrative, laboratory, pharmacy, and dental staff’ perspectives on applying AI in primary care. Furthermore, the most crucial aspect is to have the patient perspective central to improvement in health care systems.

### Conclusions

The findings from this study indicate that physicians hold a very positive perception regarding the integration of AI within primary health care services at the PHCC, foreseeing potential enhancements in health care task management and overall service delivery. This perception extends to various dimensions of family physicians’ job roles, encompassing clinical, administrative, and opportunistic health care management. The positive expectations regarding AI’s impact also extend to operational processes, anticipating improved information dissemination, enhanced health care policy formulation, optimization of treatment care pathways, more effective human resource management, and strategic financial planning processes within the PHCC. During periods of epidemics and pandemics such as the COVID-19 pandemic, the public health management role of AI is well acknowledged by family physicians for disease screening, contact tracing, risk assessment, real-time monitoring, early diagnosis, vaccine development, and formulating efficient management strategies using AI’s predictive and logistical prowess. It is important to note that AI is not perceived as a direct replacement for family physician roles, and its introduction is not anticipated to undermine the significant patient-physician relationship. Moreover, AI is not perceived as superior to the human judgment process. Although AI holds the potential to be a valuable augmentation tool for the roles of family physicians, as per their perspective, it enhances their efficiency and productivity. However, its implementation requires due diligence with a strategy that maintains the critical challenges associated with AI integration, such as concerns related to patient satisfaction, ethical considerations regarding AI accountability in cases of malpractice, and the utmost need to uphold data privacy and confidentiality, as highlighted in this study. The implementation of AI is expected to elevate care management systems, consequently enhancing the quality of care, while simultaneously streamlining costs. The perception-based insights from this study can guide future AI implementation strategies within the context of primary health care at the PHCC, helping to pave the way for a more informed and sustainable integration of this technology. This careful and patient-centered approach will be essential in unlocking the full potential of AI in improving health care delivery, while safeguarding the values and priorities that underpin the field of medicine.

## Abbreviations

AI: artificial intelligence
CT: computed tomography
DL: deep learning
ML: machine learning
PHCC: Primary Health Care Corporation

## Data Availability

All data generated or analyzed during this study are included in this published paper.
